# Efficiency evaluation of green innovation of China’s heavy pollution industries based on SBM-Lasso-Tobit model

**DOI:** 10.1371/journal.pone.0274875

**Published:** 2022-09-29

**Authors:** Chun Fu, Yanfang Li, Jing Zhang, Weiqi Min

**Affiliations:** School of Public Policy and Administration, Nanchang University, Nanchang City, Jiangxi, China; Shenzhen University, CHINA

## Abstract

Green innovation has become the goal for promoting the transformation and upgrading heavy pollution industries in the context of high-quality development, and the key factor for the success of green innovation is increasing the green innovation efficiency of heavy pollution industries. To understand the current situation of China’s industrial innovation and get out of the dilemma, we use non-expected Slacks-based model (SBM) to measure green innovation efficiency in Chinese industry, Lasso regression to screen the influencing factors of heavy pollution industries, tobit regression to study the influence degree and direction of different influencing factors on green innovation efficiency of heavy pollution industry. The results show that: (1) The green innovation efficiency of the 16 heavily polluting industries studied in this paper is generally low; (2) Coordination, green and openness all have a positive impact on the green innovation efficiency of the industry. (3) A certain degree of government scientific research support is conducive to improving the efficiency of industrial green innovation and exceeding the limit will have a restraining effect on enterprise innovation. According to the results, we put forward the corresponding policy implications.

## 1. Introduction

With the deepening of reform and opening, the economic development in China has made remarkable achievements, the Gross Domestic Product in China exceeded 90 trillion yuan for the first time in 2018, among which the development of industrial enterprises has significantly contributed to the national economy. Nevertheless, China’s industrial development is still in the state of the so-called “three high” traditional development mode of high investment, high consumption, and high emission. The report to the 19th National Congress of the Communist Party of China has proposed that because of the shift of Chinese economy from a high-speed growth stage to a high-quality development stage, green development and innovation have dominated strategic target. Therefore, Over the years, despite the contribution to economic development, traditional industries in China are also troubling for other conditions, including environmental and resource issues, where the green transformation of traditional heavy pollution industry, requiring a sophisticated approach to improve the continuous strengthening of the combination of resources and environment. The outbreak of COVID-19, air pollution related to industry were relatively reduced during public isolation [1, 2], but the long-term exposure of air pollutants will aggravate the harm of the virus to human body [3]. Due to the concern about climate change and environmental issues, green innovation is an important research topic in the world industrial upgrading and transformation is imminent [4]. In a practical point of view, green technology innovation is an important means to break through resource and environmental constraints, enhance the competitiveness of enterprises, realize the upgrading of industrial structure, and promote high-quality economic growth [5, 6].

To illustrate, the following aspects can be concluded:1. most of the heavy-polluting industries are industrial enterprises, which occupy an important position in China’s industrial system. 2. the energy and resource problems of industrial enterprises and the environmental problems caused by traditional manufacturing promote the transformation and upgrading of enterprises. 3. the way green innovation pursues has a major impact on sustainable development of all countries. As the leading industry in China, green innovation industry is estimated to dominate in Chinese most developed fields, so lowering the need for energy-using materials is essential for decreasing the environmental impact of heavy polluting industry.

Under the guidance of the concepts "green development" and "innovation-driven ", the heavy pollution industry is taken as a research object. The main research contents are as follows: Firstly, the panel data of heavy pollution industry from 2009 to 2018 are established, and the green innovation efficiency is calculated and analyzed. Secondly, the influencing factors of green innovation efficiency are screened by Lasso regression, and Tobit regression was used to study the influence degree and direction of different influencing factors based on it. Finally, Chinese heavy pollution industry is tasked with changing and improving green innovation efficiency.

## 2. Literature

### 2.1 Research on efficiency measures

Before considering the efficiency measure of green innovation, it is necessary to analyze what is efficiency. In economics, efficiency refers to the ratio of input and output of resources, which is used to achieve the goal of resource allocation and effective utilization [7]. However, as the traditional technology innovation efficiency research does not consider environmental output, but only focuses on strict economic benefits, the measurement results of traditional technology efficiency are not applicable to the current requirements of green development. To bridge this gap, the concept of green innovation efficiency has been introduced and adopted by subsequent scholar. Since the beginning of 21st century, international organizations, represented by the Organization for Economic Cooperation and Development (OECD) of the European Union (EU), have actively promoted "green innovation " and carried out systematic theoretical and practical research on it.

In the selection of innovative efficiency measurement methods, data envelopment analysis (DEA) can contain multiple inputs and outputs at the same time without setting a specific function form, which has been used by many scholars in efficiency measurement methods [8–10]. The research of other motivating factors has also been explored. For example, Fan and Zhang (2015) conducted a study focusing on the technical efficiency of carbon dioxide utilization in China through super efficiency DEA [11]. Carayannis et al. (2016) proposed a multistage DEA model that integrates the regional and stage differences by comparing innovation efficiency of 185 regions in 23 European countries [12]. Chunyang Liu et al. (2020) analyzed and calculated the efficiency of green technological innovation with SBM-DEA model, classifying the high-tech industry into different development levels for regression analysis [13]. Jiang (2020) used three-stage DEA to calculate the innovation efficiency of industrial enterprises in 30 provinces and cities in China, and analyzed its influencing factors [14]. Li (2019) analyzes the regional technological innovation and green economic efficiency based on DEA model and fuzzy evaluation [15]. Lin and Li (2022) used a two-stage series DEA model to measure the industrial innovation efficiency of 30 provinces and cities in China, and analyzed its spatial spillover effect [16]. Xu et al. (2021) evaluated the industrial entrepreneurship efficiency of 30 provinces in China based on super SBM and Malmquist index method [17].

Besides the improvement of efficiency measurement model and the study of model differences, the research object of green innovation is also related to the development of economy. At present, with the increase of environmental pressure and resource consumption, the production industries resulting in significant pollution or damage to the environment begin to carry out cleaner production, resource recovery and green transformation through technological innovation. Domestic and foreign Scholars also start to focus on the green innovation of manufacturing industry. Scholars’ research objects on innovation performance include regions [18], furniture industry [19], high patent intensive manufacturing industry [20] etc. Bartzokas (1997) introduced the characteristics of technology development in seven heavy-polluting industries and discussed the possibility of their application of clean technology [21]. Finally, it is pointed out that enterprises need to develop more "green" technology in terms of innovation of production technology.

Existing research on green innovation efficiency mainly focuses on the construction of human input indicators, capital and energy levels, as well as output indicators from the economic, innovation, and environmental perspectives, such as new product sales revenue, the number of patent applications for invention, and the comprehensive index of environmental pollution calculated from the “industrial three wastes [22–24].

### 2.2 Research on the influencing factors of innovation efficiency

Scholars define the efficiency that considers both environmental output and economic benefits as green innovation in the process of input and output of innovative resources, which is different from the traditional concept of innovation efficiency that only considers economic output [7, 18, 25]. In terms of the analysis of influencing factors of green innovation efficiency, the traditional environmental regulation [26–29], R&D investment [30], the degree of foreign participation and market competition were mainly studied [23, 31], and for environmental regulation indicators, the impact of environmental regulation on technological innovation was studied with a conclusion that environmental regulation has significant positive incentives for technological innovation in heavy-polluting industries. Some scholars have drawn different conclusions [32–34]: environmental indicators have negative inhibitory effects on green innovation efficiency, but a positive promoting effect on the green innovation efficiency. As can be seen from the literature, one of the indicators of environment-environmental regulation has both positive ecological compensation effect and cost input effect of environmental regulation on green innovation. If the compliance cost effect of environmental regulation is greater than that of ecological compensation, the total effect of environmental regulation would be negative, but this assumption is inconsistent with strict environmental regulation policies. In recent years, increasingly more scholars and researchers have studied green technology innovation policies. For example, Auld and Malet (2014) concluded that policy innovation is the prior criterion for low-carbon technologies [35]. And then in 2015, Li believed that the pollutant emission fee system has generated spatial differences in the green technology innovation drive of manufacturing enterprises [36].

Due to different research objects and methods, scholars have different understandings on the variable selection of the influencing factors of green innovation efficiency, and even the same index may cause different results, so further identification, and analysis of the influencing factors of green innovation is still one of the most important studies in academia.

Because of the lack of in-depth study on the scientific measurement of green innovation efficiency in heavy pollution industry, the absent foresight and creativity in the selection of influencing factors, as well as the unspecific identification of industry differences, this article aims to further explore the green innovation of heavy pollution industries from the three aspects: the selection of heavy polluting industries, the evaluation of innovation efficiency and the construction of green efficiency model. In this article, the SBM model considering environmental pollution is adopted to measure the efficiency of green innovation in the industry, and the optimal variables affecting different industries are screened by Lasso regression to identify the source and direction of the efficiency in different industries. Meanwhile, the panel Tobit regression model is used to measure the direction and degree of the influence of each variable on the overall green innovation efficiency, model, and algorithm.

## 3. Data and variables

### 3.1 Data resources

The data used in this paper refers to 16 heavy-polluting industries in China. Among the cited national standards for the selection of specific industries of heavy-polluting industries are Classified Management List of Environmental Protection Verification Industry of Listed Companies (No.373), Listed Companies Classification Index (2010), National Economic Industry Classification (GB/T 4754–2002) and National Economic Industry Classification (GB/T 4754–2011). After 2011, the breakdown of individual industries has also changed. For example, the unified statistical standard of oil and gas extraction industry were not selected, instead it was finally identified into 16 distinct types: Mining and Washing of coal; Mining and processing of Ferrous Metal Ores; Mining and processing of Non-Ferrous Metal Ores; processing of Food from Agricultural products; Manufacture of Textile; Manufacture of Leather, Fur, Feather and Related Products; Manufacture of Paper and Paper Products; Processing of Petroleum, coal and Other Fuels; Manufacture of Raw Chemical Materials and Chemical Products; Manufacture of Medicines; Manufacture of Chemical Fibers; Manufacture of Non-metallic Mineral Products; Smelting and Pressing of Ferrous Metals; Smelting and Pressing of Non-Ferrous Metals; Manufacture of Metal Products; Production and Supply of Electric Power and Heat Power.

The heavy polluting industry has always been well used to adapting to the advance of Chinese economy. The implementation of automation from the 1970 s onwards led to significant cost savings and improvements in the reliability and flexibility of mass production. A new challenge to heavy polluting industry is now on the horizon and, again, it comes from high pollution emissions and resource energy consumption that hinder the sustainable development of China and the world, but these can most probably be conquered within the next decades through the proposal of building innovative and green industries, especially the "leading" industry in green development-heavy pollution industry that is potentially significant to the high quality development of China. The relevant data is from China Science and Technology Statistics Yearbook, China Environment Statistics Yearbook, China Energy Statistics Yearbook, China Industrial economy Statistics Yearbook and China Statistical Yearbook from 2009 to 2018. For the lack of individual data, the data of adjacent years is added to estimate the mean value.

### 3.2 Green innovation efficiency index system

The innovation efficiency is measured by Non-expected SBM model in MATLAB software. The input-output indicators are described in detail as follows:

Input indicators focusing on human, financial and energy resources point to it as an important adaptation for innovation investment in green innovation activities. Take, for example, the heavy pollution industry. Back in the last few years, due to the human capital known as the dynamic input of innovation activities, the full-time equivalent of industry R&D personnel is selected as an alternative index. The financial support of innovation activities is represented by the internal expenditure of industry R&D funds. Energy, another essential input in the industrial industry, also plays an essential role in green innovation. This article is represented by the total energy consumption of the industry.

There are two aspects to consider expected output of innovation activities. One frequently cited motive is the number of innovations. Indeed, it is demonstrated that the number of industrial invention patents approximately represents the output of knowledge and technology. Another perspective is the quality of innovative results. The sales revenue of industrial new products can well reflect the result of innovation efficiency from the dimension of technology transformation.

Beyond these direct expected outputs, the wider implications for the non-expected output including environmental pollution are also be considered. Among these selections are industrial wastewater, waste gas, solid waste emissions, as well as the environmental pollution index calculated by entropy weight method, all of which practically improve the calculated results of green innovation efficiency. The input-output indicators are shown in Table 1.

**Table 1:** Green innovation index system of heavy pollution industry.

Primary Indicators	Secondary Indicators
Green innovation input	R&D personnel full-time equivalent
	R&D intramural expenditure capital stock
	Total energy consumed
Green innovation expected output	Patent applications
	Sales of new products
Green innovation non-expected output	Integrated Environmental Pollution Index

### 3.3 Other influence factors

Depending on the circumstances that Chinese economy is advancing so fast, this article decides to study five basic connotations of high-quality development: the green innovation efficiency of heavy pollution industry, innovation, coordination, green, openness and sharing. The influencing factors selected in this article are as follows:

Innovation level: As the first driving force of high-quality development, the innovation level of the industry supports the transformation and upgrading of industry and the promotion of green innovation efficiency. When the industry innovation level is effective, the green innovation efficiency would out-compete inefficient ones, but the reverse is likely to be true if the industry innovation level is inefficient. Such conditions are particularly troubling for the index selection, so it is proposed that the source of industry innovation has two major types, the first being internal factors. Human capital and scientific research capital investment are the two necessary elements of industry innovation. The proportion of government funds in scientific research can be used as external stimulus to express government support for industry innovation. Therefore, the proportion of internal R&D expenditure in the main business income of the industry is chosen to represent the investment of scientific research expenditure (RDE), and the expected impact is positive. The ratio of R&D personnel to the average number of employees in the industry represents human capital investment (RDP), and the expected impact is also positive. Government support (GOS), expressed as the proportion of government funds in the internal expenditure on R&D funding, is expected to be positive.

Coordination: Enterprise size (ES). The famous “Schumpeter hypothesis” has demonstrated several large enterprises that have a favorable impact on technological progress. The findings support the view that enterprise size may indeed have a beneficial effect on green and innovation, especially on heavy-polluting industries where green and innovation are costly and risky, but the framing of that finding must be carefully aligned with those large-scale enterprises that contain sufficient financial resources for innovation. In turn, small enterprises are less likely to take risks of reducing pollution emissions and energy inputs, so enterprise size is one of the influencing factors of green innovation in this article. It is expressed by the ratio of the industry’s main business income to the number of enterprise units, and the expected impact is positive.

Green: environmental regulation (ER). It is estimated in the academic research that green innovation is shaped by environmental regulation. Despite abundant existing literature and references, the research conclusion is still unanimous because environmental regulation has both ecological compensation and cost effects on green innovation, which may have heterogeneity in different regions and industries. In a green innovation study conducted by Penghui Kang, the ecological compensation effect of environmental regulation was better than that of cost effect cost. On average, the overall effect was remarkably positive, so combined with the environmental pollution index and data availability, the ratio of the annual treatment cost of wastewater and waste gas to the main business income was determined as the measurement index of environmental regulation and the expected impact was positive.

Opening: foreign direct investment (FDI). The reform and opening bring unprecedented opportunities to the rapid development of China, and even worldwide. Because of the financial support of independent innovation, foreign direct investment provides a basis for industrial green innovation, and out of the small FDI share of internal expenditure and the paucity of data, this article selects the proportion of foreign capital, Hong Kong, Macao, and Taiwan capital as an alternative index. The expected impact is positive.

Sharing: Log of Gross profits of industrial enterprises (lnPIE). In the long run, it is heavy pollution industry, either directly or through its politicians, that has the power to make the growth of Chinese economy, especially the development of regional economy, such as the cultural development, infrastructure construction and the overall rise of GDP in regions. On the contrary, the economic development of region can become the public wealth sharing and support the industry transformation and green environmental protection. The task of this index is therefore to reduce the deviation of the estimated results caused by the lack of data: for instance, the total profit of industrial enterprises selected as the logarithms represents the actual value of the index to reduce adverse effects of data fluctuations. The expected impact is positive.

To sum up, this article puts forward the following assumptions:

H1 Representing variables such as innovation level, firm investment in science and technology, personal investment and external government support have a positive effect on green innovation of heavy-polluting industry.
H2 The impact of the average enterprise scale, which reflects the degree of industry coordination, on the green innovation industry is uncertain.
H3 Environmental adjustment variable and regulation is consistent with the conclusion of Porter hypothesis.
H4 Foreign direct investment benefits the green innovation efficiency of industrial enterprises.
H5 As an alternative index of industry economic development, the total profit of industrial enterprises plays a part in green innovation.

Descriptive statistics for the above variables are shown in Table 2.

**Table 2:** Descriptive statistics of variables.

Variables	Sample size	Mean	Std.Dev	Maximum	Minimum
GIE	160	0.624	0.329	1	0.049
RDE	160	0.006	0.004	0.021	0
RDP	160	0.026	0.019	0.082	0.002
GOS	160	0.026	0.025	0.269	0.006
ES	160	4.220	4.209	22.532	0.626
ER	160	0.003	0.003	0.018	0
FDI	160	0.215	0.661	8.364	0.005
lnPIE	160	7.244	0.883	8.673	4.285

Description:Green innovation efficiency(GIE);R&D expenditure(RDE);R&D personnel(RDP);Government support (GOS);Enterprise size(ES);environmental regulation (ER);foreign direct investment (FDI);Log of Gross profits of industrial enterprises (lnPIE).

### 3.4 Research framework

**Fig 1:**
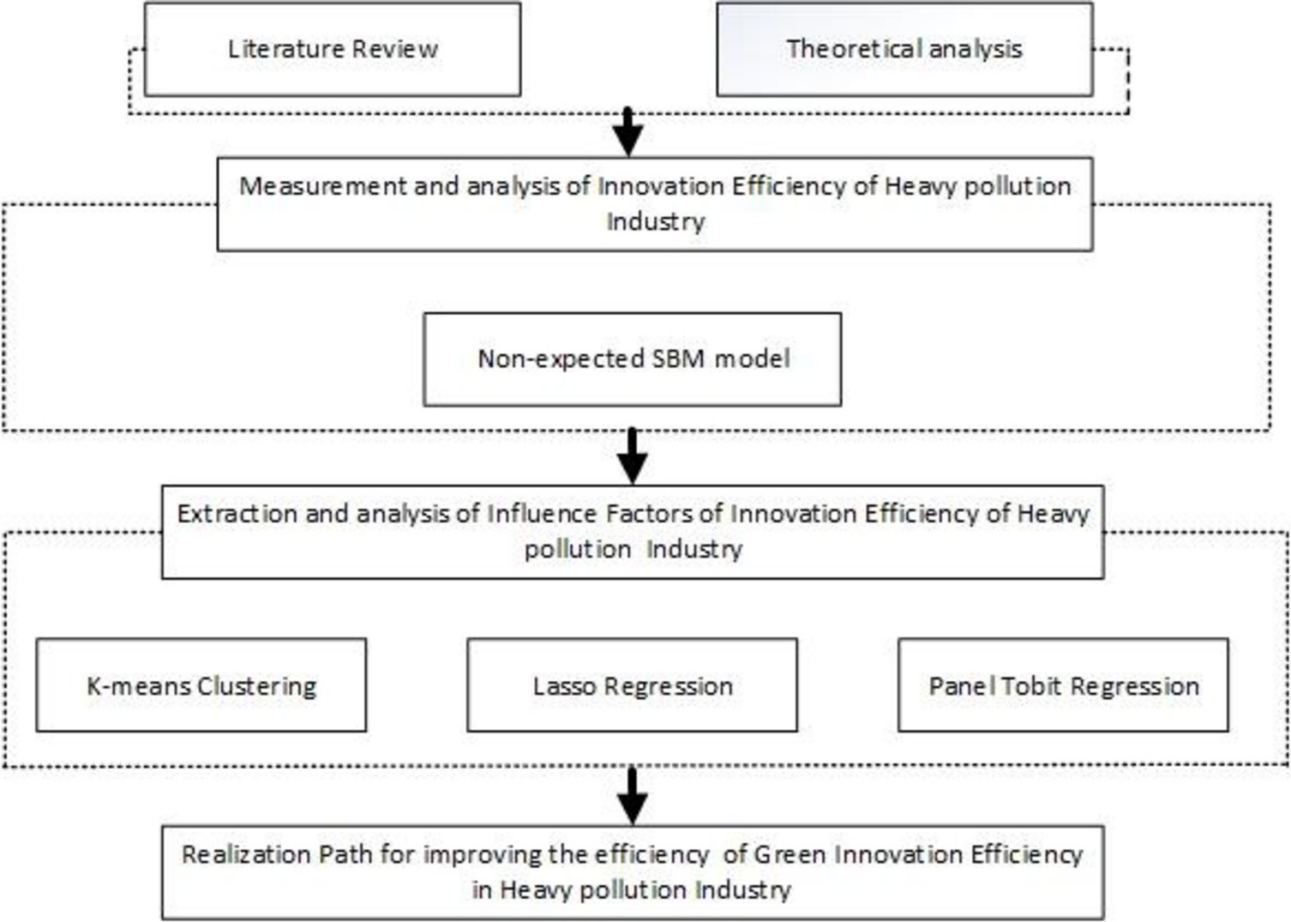
System framework of this paper.

The topic of this article is “Efficiency evaluation of green innovation of China’s heavy pollution industries: An empirical analysis based on Lasso regression”. It highlights the measurement and analysis of innovation efficiency of Heavy Pollution Industry through the earlier-20th centuries, including the green development in the now period. Of particular interest is those green innovation of heavy pollution industry to the whole economic development, so extracting and analyzing the factors affecting the efficiency of green innovation in heavy pollution industry is of great importance. The specific system framework of this paper is shown in Fig 1.

## 4. Model and algorithm

### 4.1 SBM efficiency measurement model

The traditional DEA models are mostly angular and radial. When evaluating the efficiency of certain unexpected outputs, the results of efficiency evaluation are often inaccurate due to radial selection and relaxation. To overcome this defect, Tone proposed a SBM model based on non-desired output. Unlike the traditional DEA model, the SBM model is an angle-free and diameter-free model by adding relaxation variables into the objective function.

Each industry is regarded as an independent decision-making unit (DMU) of the production system, and each decision-making unit contains three vectors: the corresponding input element, the expected output, and the unexpected output. The model is calculated and illustrated as follows:

$$\rho =\min\frac{1‐\frac{1}{m}\sum_{i=1}^{m}\frac{s_{1}^{-}}{x_{i0}}}{1+\frac{1}{s_{1}+s_{2}}(\sum_{r=1}^{s_{1}}\frac{s_{r}^{a}}{y_{r0}^{a}}+\sum_{r=1}^{s_{2}}\frac{s_{r}^{b}}{y_{r0}^{b}})}$$ (1)

$$\mathrm{st}.\left\{ \begin{array}{l} X_{0}=X\lambda +S^{-}\\ Y_{0}^{a}\lambda =Y^{a}\lambda -S^{a}\\ Y_{0}^{b}=Y^{b}\lambda +S^{b}\\ s^{-}>0,s^{a}>0,s^{b}>0,\lambda >0 \end{array}\right.$$

Where *ρ* refers to the target planning value, *μλ* represents the planning decision variable, s^-^、s^a^、s^b^ is a relaxed variable vector. For any decision unit, the value of the *ρ* is in the range of 0 to 1. It is valid only if its objective function value is 1, and if in other cases, the result is the opposite, so it is necessary to adjust the ratio of input and output for the improvement of effectiveness.

### 4.2 Lasso regression algorithm

In general, in the regression of factors affecting a variable, the following linear regression model is first constructed:

$$Y∼\beta_{1}x_{1}+\beta_{2}x_{2}+\beta_{3}x_{3}+\cdots +\beta_{i}x_{i}$$ (2)

In Formula (2), Y is the explained variable, X = (x1, x2,…, xi) is the matrix of explanatory variables of dimension; *ε* is the vector of stochastic errors; *β* is the coefficient vector of regression model. And then the coefficients of independent variables are further estimated by least square method.

However, if there is a correlation between variables, the ordinary least square method would have a bad performance in the accuracy and complexity of prediction. Therefore, Tibshirani first proposed a new method called lasso regression to solve multiple collinearity and variable selection. Lasso regression is a compression estimation method, which introduces a penalty function in the regression model and compresses the regression coefficient of fuzzy variables to 0. Its core idea is minimizing the residual squared sum by imposing constraints on the L1 norm of the regression coefficients. The contents are as follows:

$${\hat{\beta }}_{lasso}=\underset{\beta \in R}{\arg\;\min}\left\{\frac{1}{n}{\left\|Y-X\beta \right\|}_{2}^{2}+\lambda {\left\|\beta \right\|}_{1}\right\},\lambda >0.$$ (3)

In Formula (3), *λ* is the penalty parameter. It can reduce the deviation by reducing the coefficient *β* and automatically select the variable when estimating components of the coefficient vector *β*.

### 4.3 Tobit regression algorithm

As the efficiency values calculated by the SBM model based on non-expected outputs are discrete and the values are between 0~1, the regression coefficient analysis in the ordinary least square method will lead to deviation and inconsistency in the parameter estimates value [37]. In 1958, Tobin proposed the maximum likelihood estimation method to intercept regression model (truncated regression model) [38] (Frenzen and Davis, 1990). The model is set as:

$$Y=\{\begin{array}{l} Y^{*}==\beta X+\mu_{i}\\ 0 \end{array}\;\begin{array}{c} Y^{*}>0\\ Y^{*}\leq 0 \end{array}$$ (4)

In Formula (4), The Y* is truncated dependent variable vector, the Y is green innovation efficiency value vector, the X is independent variable vector, and *β* is regression coefficient vector, *μ*_i_ is an error term, and *μ*_i_~*N*(0,*σ*^2^).

Like the Tobit model, Tobit are nonlinear models, and the estimator *β* cannot be directly used as the marginal effect Y the explained variable, but from Formula (4) it can be seen that *β* can be used as the marginal effect of the truncated variable Y* because they are linear. The hypothesis test of the Tobit model is realized by the likelihood ratio test (Likelihood Ratio Test, LR), the original hypothesis of which is: *H*_0_: *β* = *β*_0_

$$LR\;statistics\;are:\;LR=-2(\ln L_{r}‐\ln L_{u})∼\chi^{2}(j).$$

## 5. Empirical analysis

### 5.1 Measurement of green innovation efficiency

As can be seen from Table 3, the overall green innovation efficiency of most industries has experienced a curve growth trend, with the overall average green innovation efficiency rising from 0.521 in 2009 to 0.718 in 2018. The green innovation efficiency of Manufacture of Leather, Fur, Feather and Related Products and Manufacture of Medicines industries was always stable and effective. In 2010, the innovation efficiency of Manufacture of Chemical Fibers and Production and Supply of Electric Power and Heat Power industries reached 0.571 and 0.140 respectively, both of whose green innovation efficiency reached 1 in the remaining research period and the overall mean value reached 0,957 and 0.914 respectively. It is worth noting that the average green innovation efficiency of Mining and washing of coal, Mining and processing of Ferrous Metal Ores, Mining and processing of Non-Ferrous Metal Ores, Manufacture of Raw Chemical Materials and Chemical Products and Manufacture of Non-metallic Mineral Products is less than 0.5, which indicates an inefficient green innovation level, therefore, industry innovation and industrial upgrading are related to high quality development.

**Table 3:** Green innovation efficiency of China’s heavy-pollution industries in 2009–2018.

Industries/Years	2009	2010	2011	2012	2013	2014	2015	2016	2017	2018	Mean
Mining and washing of coal	0.131	0.079	0.123	0.155	0.163	0.145	0.129	0.114	0.136	0.250	0.142
Mining and processing of Ferrous Metal Ores	0.049	0.112	0.226	0.285	0.259	0.308	0.205	0.216	0.212	0.338	0.221
Mining and processing of Non-Ferrous Metal Ores	0.201	0.132	0.121	0.358	0.304	0.182	0.237	0.261	0.262	0.311	0.237
processing of Food from Agricultural products	0.313	0.439	0.425	0.563	0.486	0.529	0.600	0.585	0.605	0.664	0.521
Manufacture of Textile	0.541	1.000	0.603	1.000	1.000	1.000	1.000	1.000	0.922	0.617	0.868
Manufacture of Leather, Fur, Feather and Related Products	1.000	1.000	1.000	1.000	1.000	1.000	1.000	1.000	1.000	1.000	1.000
Manufacture of Paper and Paper Products	0.302	1.000	0.400	0.464	0.467	0.536	0.514	0.667	1.000	0.709	0.606
Processing of Petroleum, coal, and Other Fuels	0.306	0.256	0.387	1.000	1.000	1.000	1.000	1.000	1.000	1.000	0.795
Manufacture of Raw Chemical Materials and Chemical Products	0.242	0.284	0.319	0.395	0.402	0.433	0.428	0.472	0.595	0.570	0.414
Manufacture of Medicines	1.000	1.000	1.000	1.000	1.000	1.000	1.000	1.000	1.000	1.000	1.000
Manufacture of Chemical Fibers	1.000	0.571	1.000	1.000	1.000	1.000	1.000	1.000	1.000	1.000	0.957
Manufacture of Non-metallic Mineral Products	0.335	0.353	0.272	0.326	0.362	0.364	0.347	0.372	0.361	0.490	0.358
Smelting and Pressing of Ferrous Metals	1.000	1.000	0.389	0.357	0.362	0.370	0.342	0.388	0.495	0.535	0.524
Smelting and Pressing of Non-Ferrous Metals	0.336	0.528	0.523	0.455	0.546	0.714	0.677	0.817	1.000	1.000	0.660
Manufacture of Metal Products	0.586	1.000	1.000	0.655	0.595	0.607	0.606	0.617	1.000	1.000	0.767
Production and Supply of Electric Power and Heat Power	1.000	0.140	1.000	1.000	1.000	1.000	1.000	1.000	1.000	1.000	0.914
Cluster 1	0.908	0.742	0.921	1.000	1.000	1.000	1.000	1.000	0.984	0.923	0.948
Cluster 2	0.192	0.192	0.212	0.304	0.298	0.286	0.269	0.287	0.313	0.392	0.275
Cluster 3	0.474	0.704	0.521	0.582	0.576	0.626	0.623	0.679	0.850	0.818	0.645
Industries/Mean	0.521	0.556	0.549	0.626	0.622	0.637	0.630	0.657	0.724	0.718	0.624

There is a fluctuation on the efficiency of technological innovation in other industries, among which the textile manufacturing industry was the most obvious one. Its maximum efficiency value reached 1 within 6 years but fell from 0.922 to 0.664 in one year. Similarly, the manufacture of paper and paper products started at 0.302 with an upward trend, reaching an effective state in 2017, after which it showed a rapid downward trend. Processing of petroleum, coal and other Fuels and Smelting and pressing of non-ferrous metals could be two examples in the success of innovation and green transformation, with green innovation capacity continuing growing, while the figure of smelting and pressing of ferrous metals showed an opposite trend, as this industry has difficulties in promoting green innovation. In terms of time series, the efficiency value of industry has been rising almost every year since 2013, and eventually reached the effective level of the industry during the research period.

In the remaining industries, Mining and washing of coal, Mining and processing of Ferrous Metal Ores, Mining and processing of Non-Ferrous Metal Ores, processing of Food from Agricultural product, Manufacture of Raw Chemical Materials and Chemical Product, Manufacture of Non-metallic Mineral Products are fluctuate a little, in other words, the final and initial efficiency values of these industries barely change.

### 5.2 K-means clustering

**Fig 2:**
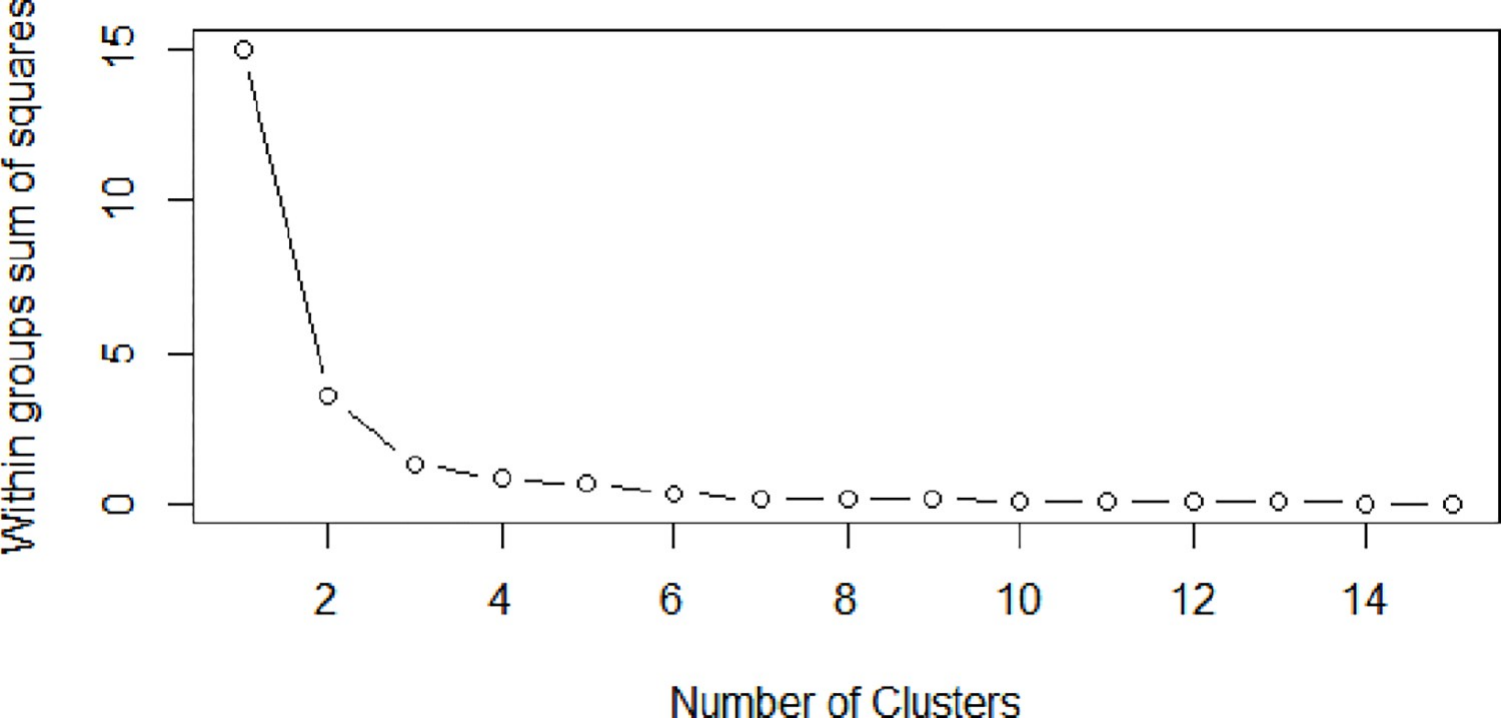
Clustering results of 16 heavy polluting industries under k-means.

Several developing countries are characterized by uneven development among different regions, so to better understand the development level of green innovation in heavily polluting industries, 16 heavy-polluting industries were classified by K-means clustering according to the average green innovation efficiency. The results show that the green innovation level of these industries can be divided into three groups: effective, ineffective, and extremely ineffective. The clustering results are shown in Fig 2 which illustrates that the sum of squares decreases within the group from type 1 to type 3, but the subsequent decline is slow. The results in Table 4 show 16 heavy pollution industries divided into three categories, with sample size 5,5,6 respectively.

**Table 4:** Clustering division of 16 heavy polluting industries.

Cluster 1 Manufacture of Textile; Manufacture of Leather, Fur, Feather and Related Products; Manufacture of Medicines; Manufacture of Chemical Fibers; Production and Supply of Electric Power and Heat Power
Cluster 2 Mining and washing of coal; Mining and processing of Ferrous Metal Ores; Mining and processing of Non-Ferrous Metal Ores; Manufacture of Raw Chemical Materials and Chemical Products; Manufacture of Non-metallic Mineral Products
Cluster 3 processing of Food from Agricultural products; Manufacture of Paper and Paper Products; Processing of Petroleum, coal and Other Fuels; Smelting and Pressing of Ferrous Metals; Smelting and Pressing of Non-Ferrous Metals; Manufacture of Metal Products

Table 4 shows that between 2009 and 2018, the Manufacture of Textile, Manufacture of Leather, Fur, Feather and Related Products, Manufacture of Medicines, Manufacture of Chemical Fibers, Production and Supply of Electric Power and Heat Power ranked first in the cluster type, with the green innovation efficiency value at 0.948, which was higher than that of the average level at 0.624, while the green innovation efficiency of the second cluster, including Mining and washing of coal, Mining and processing of Ferrous Metal Ores, Mining and processing of Non-Ferrous Metal Ores, Manufacture of Raw Chemical Materials and Chemical Products, and Manufacture of Non-metallic Mineral Products, was far lower than that of the average level. The efficiency value of green innovation in the third category is 0.645, which is basically consistent with the average level.

### 5.3 Lasso regression

**Fig 3:**
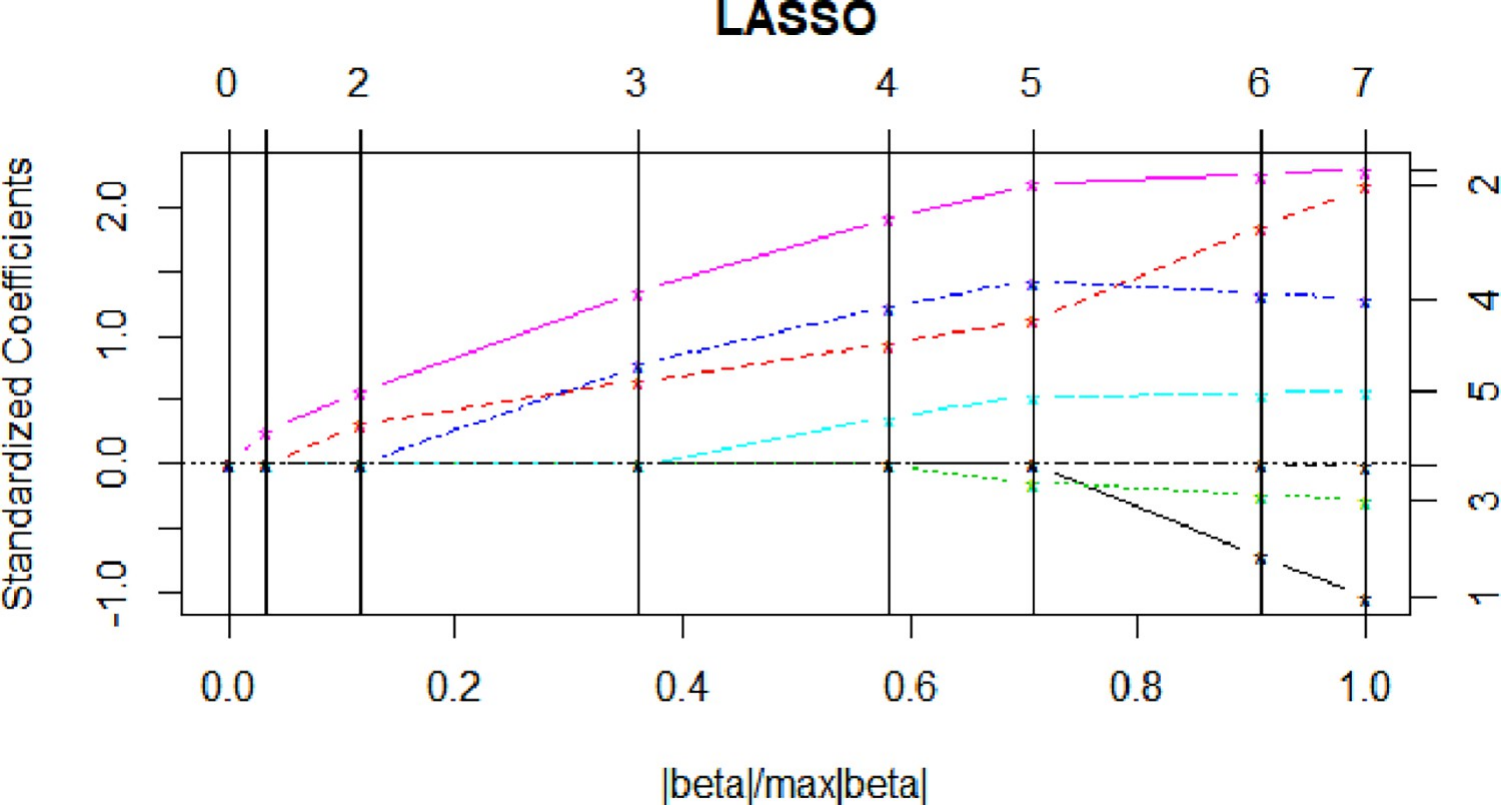
Components variation of the coefficient vector within the industry scope.

According to the level of this penalty coefficient *λ*, the algorithm sets the regression coefficient of “unimportant” variables to 0, so as to achieve the purpose of variable selection. Take 16 specific industries as example. The variable selection of each cluster industry is similar, and the different components of the coefficient vector within the industry scope are shown in Fig 3.

As can be seen from the vertical line in Fig 3, the number of iterations corresponding to Lasso, where the independent variable whose coefficient value is not 0 is the selected independent variable. With the increase of non-zero coefficient from left to right, more variables are selected into the model. The corresponding CP value changes are shown in Table 5. As can be seen from Table 5, the minimum CP value at 5.577, corresponding to step 5, is the independent variable coefficient of the row corresponding to the number of iterations. The variable corresponding to the coefficients is the optimal variables selected by Lasso regression.

**Table 5:** CP value changes corresponding to Lasso.

step	0	1	2	3	4	5	6	7
CP	93.172	84.192	64.445	23.965	7.245	5.577	6.152	8.000

Lasso regression method is chosen to analyze the key factors that affect the green innovation efficiency of heavy pollution industry under the background of high-quality development in China. Through the establishment of Lasso regression method, the optimal variable coefficients of each industry selected are shown in Table 6.

**Table 6:** Lasso regression coefficient of industries in different cluster.

Variables	All industries	Cluster 1	Cluster 2	Cluster 3
RDE	0	-88.904	50.280	12.065
RDP	4.773	38.456	-21.886	0
GOS	-0.460	2.858	1.482	0
ES	0.027	0	0.230	0
ER	13.112	-11.425	-48.516	0
FDI	1.331	0.127	4.751	0
lnPIE	0	-0.285	-0.154	0.143

The results of Lasso regression show that when the 16 industries as a whole is regressed, RDE and lnPIE variable coefficients are both 0. RDE and InPIE have their own effects in the three industry clusters. RD personnel input was only significantly limited in cluster 2, and positive assumptions that met expectations were achieved in both the industry and cluster 1. The governmental support of scientific research did not achieve the expected positive impact, on the contrary, the overall innovation of this industry is negative. Enterprise size (ES) has a weak positive effect on all industries and cluster 2. The coefficients of clusters 1 and 3 are both 0, which means in the two clusters, this variable can be rounded off. Environmental regulation (ER) promotes green innovation of the whole industry and has obvious obstacles to clusters 1 and 2. Foreign direct investment (FDI) has a positive effect on cluster 1, cluster 2 and all industries, but has no impact on cluster 3.

### 5.4 Tobit regression

The above regression results show that the proportion of internal expenditure (RDE) is one of the indicators of innovation level for 16 specific industries, after eliminating the coefficient of 0, while in the panel Tobit regression, the influence of this factor on the efficiency of green innovation was not considered. In addition, the total profits (lnPIE) of industrial enterprises are also excluded from the model. Factors that affect the selection of lasso regression include: Manpower Investment (RDP), Government Research Support (GOS), Enterprise Size (ES), Environmental Regulation (ER) and Foreign Direct Investment (FDI).

Panel Tobit regression was used to further analyze the variables selected by Lasso regression. This article discussed the influence degree of each dimension influencing factors on green innovation efficiency of heavy pollution industry and as well as studying the different effects of each factor, the influence law of each influencing factor on technological innovation efficiency in China was also revealed. The panel regression results based on the whole industry are shown in Table 7.

**Table 7:** Panel Tobit regression results in 16 heavily polluted industries.

GIE	Coef.	Std. Err.	t	Prob.
RDP	6.700***	1.954	3.43	0.001
GOS	-0.805	1.306	-0.62	0.538
ES	0.043***	0.010	4.14	0.000
ER	14.735	10.836	1.36	0.176
FDI	1.855***	0.286	6.49	0.000

Note: Above regression result is calculated by stata14.0.

***, **and *are significant at the levels of 1%, 5% and 10%, respectively.

As shown in the figure, the five independent variables can be divided into two categories: significant variable and non-significant variable.

The regression coefficient of innovation manpower input (RDP) and foreign direct investment (FDI) are both positive, with P values of 0.001 and 0.000 respectively, which suggests that the investment of scientific research personnel and foreign direct investment are both conducive to green industrial innovation, promoting the innovation level of heavy pollution industry and high-quality economic development. Besides, the regression results of enterprise size (ES) are equally significant, which means that larger enterprises are more likely to take risks and are more capable of assuming the enterprise innovation hypothesis, which happens to coincide with the conclusion of domestic scholar called Wu Chao.

Government research support (GOS) should have a positive impact on industrial green innovation, but the results show that its impact on green innovation is small, which is inconsistent with the hypothesis. In addition, environmental regulation (ER) also has a slight positive effect on enterprise innovation, with 14.735 regression coefficient.

Each factor is analyzed as follow:

The contribution of scientific research personnel (RDP) significantly improve the green innovation efficiency of heavy pollution industries. The increase of the proportion of scientific research personnel directly affects the allocation efficiency of production factors, improves the efficiency of R & D heavy-polluting industry innovation, and promotes the new normal of Chinese secondary industry upgrading and transformation.

Enterprise scale (ES). The green innovation of enterprises requires funds to purchase advanced equipment or green materials and talents to produce the "green" products, through scientific research, which is conducive to environmental protection, resource saving, as well as the healthy growth of Chinese economy, but the achievement of enterprise green innovation should be based on the enterprise itself. In other words, as the carrier of green innovation, the larger-scale enterprises are more likely to support the risks and uncertainties brought by green innovation, which further proves the correctness of the current enterprise merger and acquisition.

Since the reform and opening up, Chinese economy has developed rapidly with a rising international status, which proves that Chinese opening up is for the whole population, and in terms of industries, opening up means "goods out" and "investment in ", so foreign direct investment (FDI) plays a significant role in promoting green innovation of the whole industry because it can not only bring a large amount of capital to support innovation activities, but also directly bring advanced technologies to the industry, which would benefit the sustainable and healthy development of heavy pollution industry.

Government support (GOS) is damaging to the improvement of green innovation efficiency, which means the higher the efficiency of green innovation is, the greater the negative impact would be. The possible reason for this result is that government investment may squeeze out the R&D investment of the enterprise, and lead to the insufficient R&D investment, which to some extent inhibits the innovation of the enterprise and makes the return result negative, even though it may effectively solve the problem of insufficient funds for enterprise innovation and R&D activities, promoting enterprises to carry out efficient innovative activities.

Environmental Regulation (ER).The research on environmental regulation and green innovation efficiency has always been a hot topic in academic circles. The research results in this article further confirm the correctness of Porter’s hypothesis [39], that is, the pollution emission control of industrial waste has obvious positive significance for the green development of enterprises.

## 6. Conclusions and policy implications

The main results are as follows: (1) In the calculation of innovation efficiency, only 2 of the 16 heavily polluting industries are Manufacture of Leather,Fur,Feather and Related Products and Manufacture of Medicines, while the other 14 industries are all non-effective industries, so the green innovation efficiency can be optimized by adjusting the input. (2)In the K-means clustering, 16 heavily polluting industries are divided into three categories, but in the careful analysis of the mean value of innovation efficiency data, it can be found that the clustering method is based on the mean value of industry innovation efficiency, but due to the randomness of this clustering method, results generated by each clustering are inconsistent, so the same data in different articles may produce inconsistent result retelling. (3) The coefficient of variable RDE and lnPIE is 0, which means the two variables are eliminated in the choice of variables, and they may have little effect on industrial green innovation. Regarding the three clusters, the internal expenditure (RDE) of the industry has obvious inhibitory effect on cluster 1, while the effect in cluster 2 and 3 is opposite. The logarithm (lnPIE) of the total profit of industrial enterprises has a negative hindrance effect on cluster 1 and 2, and only cluster 3 accords with the expected positive hypothesis. (4) The influencing factors are divided according to the five basic connotations of high-quality development in China. The human input and government support that reflect the innovation level have a significant positive effect and a certain degree of inhibition on green innovation level of the industry. Firm size (ES), which represents the degree of coordination, has a significant positive effect on the improvement of green innovation efficiency. It indicates that green environmental regulation (ER) is an effective driving force for the improvement of innovation efficiency in heavy polluting industries. Openness (FDI) has achieved the expected improvement in the efficiency of green innovation in the industry.

In view of the conclusion of this article, the improvement of green innovation efficiency in heavily polluting industries takes four policy suggestions: At a very basic level, the investment of scientific research personnel should be improved for the development and application of green technology. Critically, recent research supports the idea that green technology can facilitate the transformation and upgrading of enterprises in heavily polluting industries. Therefore, the government’s role in increasing the investment of R & D personnel of special technologies such as cleaner production, resource saving and recycling, varies as a function of accelerating the popularization and application of green technology to realize the green transformation of heavy pollution industries. (2) environmental regulation policy and the green environmental protection consciousness of enterprises should be raised. In the more direct form of environmental regulation, the green efficiency promotion mode, the enforcement of environmental regulation policies, the management mechanisms of industry examination and approval, pollution emission, cleaner production audit and industry elimination, and promote the formation of green environmental protection consciousness of enterprises in heavy pollution industries by means of mechanism binding force. (3) the mergers and acquisitions of small and medium-sized enterprises should be supported to increase their risk-tearing capacity for green innovation. Enterprise scale has an obvious promoting effect on industrial green innovation. Its increase not only improves the scale economy of enterprises, but also increases the opportunity for the implementation of green innovation. (4) introduced foreign direct investment and the expansion of the absorption of capital technology in heavy pollution industries should be supported. In addition to increasing the support for scientific research funds of enterprises, foreign investment also brings them foreign advanced technical experience, so that foreign investment can exist in enterprises with certain knowledge reserves.

In recent decades, due to the different effects of environmental regulation, foreign direct investment, a certain degree of government support and other relevant policies, the importance of enterprise size has never disappeared. It was once regarded as a trivial and even negative thing in contrast to "green and innovation", but we should not ignore its benefits and its fundamental contribution to human achievements in the green innovation efficiency of the heavily polluting industries targeted by the complementary portfolio policy. In general, environmental regulatory policies and strong government scientific research support are the basic elements to improve the green efficiency of large-scale heavy pollution industries with the environment as the center, and the introduction of foreign direct investment and sufficient scientific researchers is the way for green and innovation to obtain more targeted experience. In either case, small and medium-sized heavy polluting enterprises should actively participate, especially foreign direct investment. This paper measures the green innovation efficiency of China’s heavy pollution industry and describes the impact of different influencing factors on the green innovation of the pollution industry, to find out the different influencing factors of the green innovation efficiency of the heavy pollution industry and strive to improve the level of green innovation in China. Subsequent research can update the influencing factors according to the background of the new era to get more accurate research results.

## References

[1] Ali G, Abbas S, Qamer FM, Wong MS, Rasul G, Irteza SM, et al. Environmental impacts of shifts in energy, emissions, and urban heat island during the COVID-19 lockdown across Pakistan. J Clean Prod. 2021;291: 125806. doi: 10.1016/j.jclepro.2021.125806 PMC9759398 36569464

[2] Ju MJ, Oh J, Choi YH. Changes in air pollution levels after COVID-19 outbreak in Korea. Sci Total Environ. 2021;750: 141521. doi: 10.1016/j.scitotenv.2020.141521 32829258 PMC7402377

[3] Marquès M, Domingo JL. Positive association between outdoor air pollution and the incidence and severity of COVID-19. A review of the recent scientific evidences. Environ Res. 2022;203. doi: 10.1016/j.envres.2021.111930 34425111 PMC8378989

[4] Luo X, Zhang W. Green innovation efficiency: a threshold effect of research and development. Clean Technol Environ Policy. 2021;23: 285–298. doi: 10.1007/s10098-020-01977-x

[5] Ze-Lei X, Ru-Guo F, Xin-Ya D. Measurement and Convergence of China’s Regional Innovation Capability. Sci Technol Soc. 2019;24: 1–28. doi: 10.1177/0971721818806079

[6] Du JL, Liu Y, Diao WX. Assessing regional differences in green innovation efficiency of industrial enterprises in China. Int J Environ Res Public Health. 2019;16. doi: 10.3390/ijerph16060940 30875986 PMC6466068

[7] Luo Q, Miao C, Sun L, Meng X, Duan M. Efficiency evaluation of green technology innovation of China’s strategic emerging industries: An empirical analysis based on Malmquist-data envelopment analysis index. J Clean Prod. 2019;238: 117782. doi: 10.1016/j.jclepro.2019.117782

[8] Xi P, Zhu Q. Analysis of China’s regional energy utilization and environment protection efficiency based on the DEA-SBM model. Int J Bus Anal. 2017;4: 1–19. doi: 10.4018/IJBAN.2017040101

[9] Li D, Zeng T. Are China’s intensive pollution industries greening? An analysis based on green innovation efficiency. J Clean Prod. 2020;259: 120901. doi: 10.1016/j.jclepro.2020.120901

[10] Long R, Guo H, Zheng D, Chang R, Na S. Research on the Measurement, Evolution, and Driving Factors of Green Innovation Efficiency in Yangtze River Economic Belt: A Super-SBM and Spatial Durbin Model. Complexity. 2020;2020. doi: 10.1155/2020/8094247

[11] Fan JL, Zhang X, Zhang J, Peng S. Efficiency evaluation of CO2 utilization technologies in China: A super-efficiency DEA analysis based on expert survey. J CO2 Util. 2015;11: 54–62. doi: 10.1016/j.jcou.2015.01.004

[12] Carayannis EG, Grigoroudis E, Goletsis Y. A multilevel and multistage efficiency evaluation of innovation systems: A multiobjective DEA approach. Expert Syst Appl. 2016;62: 63–80. doi: 10.1016/j.eswa.2016.06.017

[13] Liu C, Gao X, Ma W, Chen X. Research on regional differences and influencing factors of green technology innovation efficiency of China’s high-tech industry. J Comput Appl Math. 2020;369: 112597. doi: 10.1016/j.cam.2019.112597

[14] Yanting J. The Measurement of Innovation Efficiency of Chinese Industrial Enterprises. Value Eng. 2020;39: 123–125. doi: 10.14018/j.cnki.cn13-1085/n.2020.04.054

[15] Li Q. Regional technological innovation and green economic efficiency based on DEA model and fuzzy evaluation. J Intell Fuzzy Syst. 2019;37: 6415–6425. doi: 10.3233/JIFS-179220

[16] Runtian L, Bizhen L. Measurement of industrial innovation efficiency and analysis of spatial spillover effect in China. Stat Decis. 2022;38: 97–101. doi: 10.13546/j.cnki.tjyjc.2022.07.019

[17] Wei X, Zhiru L, Yuanyuan Z, Huibin S. Evaluation of China’s industrial innovation efficiency based on supre-SBM model and Malmquist index. Macroecon Manag. 2021;5: 55–68. doi: 10.16304/j.cnki.11-3952/f.2021.05.006

[18] Qun Y, Yue C. Research on regional differences and causes of green innovation efficiency in China. Jiangsu Soc Sci. 2016;2: 64–69. doi: 10.13858/j.cnki.cn32-1312/c.2016.02.009

[19] Sellitto MA, Camfield CG, Buzuku S. Green innovation and competitive advantages in a furniture industrial cluster: A survey and structural model. Sustain Prod Consum. 2020;23: 94–104. doi: 10.1016/j.spc.2020.04.007

[20] Jian H, Heng C. Research on the performance and driving factors of green transformation of technological innovation in China’s high patent intensive manufacturing industry. Manag Review. 2018;30: 132–142. doi: 10.14120/j.cnki.cn11-5057/f.2018.04.006

[21] Bartzokas A, Yarime M. Technology Trends in Pollution-Intensive Industries: A Review of Sectoral Trends. Discuss Pap Ser. 1997;9706: 56.

[22] Zhijun F. Research on Industrial Enterprise’s Innovation Efficiency in China Based on Regional Comparison. China Sci Technol Forum. 2013; 82–88. doi: 10.13580/j.cnki.fstc.2013.02.015

[23] Ling L, Xiaohua X. Research on green innovation efficiency and influencing factors of pollution intensive industries. Res Social with Chinese Charact. 2018; 83–88.

[24] Neng S, Jinjin Z. Research on the efficiency of green innovation and the mechanism of key factors in China from the perspective of technological heterogeneity: Based on hybrid DEA and structural equation model. J Ind Eng Manag. 2018;32: 46–53.

[25] Chen J, Cheng J, Dai S. Regional eco-innovation in China: An analysis of eco-innovation levels and influencing factors. J Clean Prod. 2017;153: 1–14. doi: 10.1016/j.jclepro.2017.03.141

[26] Zhou Y, Zhu S, He C. How do environmental regulations affect industrial dynamics? Evidence from China’s pollution-intensive industries. Habitat Int. 2017;60: 10–18. doi: 10.1016/j.habitatint.2016.12.002

[27] Ke J, Yuhua T. Impact of China ‘ s Environmental Regulation on the Industrial Technological Innovation, Based on the Panel Data of 20 Pollution Intensive Industry of China. Ecol Econ. 2014;30: 90–93.

[28] Xie R hui, Yuan Y jun, Huang J jing. Different Types of Environmental Regulations and Heterogeneous Influence on “Green” Productivity: Evidence from China. Ecol Econ. 2017;132: 104–112. doi: 10.1016/j.ecolecon.2016.10.019

[29] Liu Y, Zhu J, Li EY, Meng Z, Song Y. Environmental regulation, green technological innovation, and eco-efficiency: The case of Yangtze river economic belt in China. Technol Forecast Soc Change. 2020;155: 119993. doi: 10.1016/j.techfore.2020.119993

[30] Wong SKS. Environmental requirements, knowledge sharing and green innovation: Empirical evidence from the electronics industry in China. Bus Strateg Environ. 2013;22: 321–338. doi: 10.1002/bse.1746

[31] Ming Y, Xiaoman C. Spatial and temporal differentiation of urban green innovation efficiency in the Yangtze River economic belt and its influencing factors. Urban Probl. 2018;7: 44–68.

[32] Yan L, Ping C. Research on the threshold effect of environmental regulation on the improvement of China’s industrial green innovation efficiency. J Northeast Univ Sci. 2018;20: 147–154.

[33] Xiaojun D, Yating F. Research on the relationship between environmental regulation and green innovation in China empirical analysis based on meta analysis. Price theory Pract. 2018;6: 34–37. doi: 10.19851/j.cnki.cn11-1010/f.2018.06.009

[34] Song M, Wang S, Sun J. Environmental regulations, staff quality, green technology, R&D efficiency, and profit in manufacturing. Technol Forecast Soc Change. 2018;133: 1–14. doi: 10.1016/j.techfore.2018.04.020

[35] Auld G, Mallett A, Burlica B, Nolan-Poupart F, Slater R. Evaluating the effects of policy innovations: Lessons from a systematic review of policies promoting low-carbon technology. Glob Environ Chang. 2014;29: 444–458. doi: 10.1016/j.gloenvcha.2014.03.002

[36] Wanhong L. Spatial metrological test of green technology innovation driven by sewage charge system—a case study of 29 provincial manufacturing industries. Sci Res Manag. 2015;36: 1–9. doi: 10.19571/j.cnki.1000-2995.2015.06.001

[37] Bone PF. Word-of-mouth effects on short-term and long-term product judgments. J Bus Res. 1995;32: 213–223. doi: 10.1016/0148-2963(94)00047-I

[38] Frenzen JK, Davis HL. Purchasing Behavior in Embedded Markets. J Consum Res. 1990;17: 1. doi: 10.1086/208532

[39] Porter ME. America’s green strategy. Sci Am. 1991;264: 168. Available: http://www.citeulike.org/group/2016/article/1096884

